# Nanotechnology-Driven Strategy Against SARS-CoV-2: Pluronic F127-Based Nanomicelles with or Without Atazanavir Reduce Viral Replication in Calu-3 Cells

**DOI:** 10.3390/v17040518

**Published:** 2025-04-01

**Authors:** Eduardo Ricci-Junior, Alice Santos Rosa, Tatielle do Nascimento, Ralph Santos-Oliveira, Marcos Alexandre Nunes da Silva, Debora Ferreira Barreto-Vieira, Luísa Tozatto Batista, Giovanna Barbosa da Conceição, Tayane Alvites Nunes Quintão, Vivian Neuza Santos Ferreira, Milene Dias Miranda

**Affiliations:** 1 Galenic Development Laboratory, University Pharmacy, Universidade Federal de Rio de Janeiro, Rio de Janeiro 21941-902, Brazil; tatiellenascimento94@gmail.com; 2 Laboratory of Morphology and Viral Morphogenesis, Oswaldo Cruz Institute, Oswaldo Cruz Foundation, Rio de Janeiro 21040-900, Brazil; alicerosa@aluno.fiocruz.br (A.S.R.); marquinhosans@gmail.com (M.A.N.d.S.); barreto@ioc.fiocruz.br (D.F.B.-V.); luisatozbatista@gmail.com (L.T.B.); giovannaconceicao@id.uff.br (G.B.d.C.); tayanequintao1@gmail.com (T.A.N.Q.); vivian.ferreira@ioc.fiocruz.br (V.N.S.F.); 3 Programa de Pós-graduação em Biologia Celular e Molecular, Instituto Oswaldo Cruz, Fundação Oswaldo Cruz, Rio de Janeiro 21040-900, Brazil; 4 Laboratory of Nanoradiopharmacy and Synthesis of Novel Radiopharmaceuticals, Nuclear Engineering Institute, Rio de Janeiro 21941-906, Brazil; presidenciaradiofarmacia@gmail.com; 5 Laboratory of Nanoradiopharmacy and Radiopharmaceuticals, Zona Oeste State University, Rio de Janeiro 21941-906, Brazil; 6 Programa de Pós-graduação em Medicina Tropical, Instituto Oswaldo Cruz, Fundação Oswaldo Cruz, Rio de Janeiro 21040-900, Brazil

**Keywords:** SARS-CoV-2, Calu-3 cells, antiviral activity, nanotechnology, polymeric micelles, poloxamer, Pluronic F127, atazanavir (ATV)

## Abstract

Despite extensive efforts, no highly effective antiviral molecule exists for treating moderate and severe COVID-19. Nanotechnology has emerged as a promising approach for developing novel drug delivery systems to enhance antiviral efficacy. Among these, polymeric nanomicelles improve the solubility, bioavailability, and cellular uptake of therapeutic agents. In this study, Pluronic F127-based nanomicelles were developed and evaluated for their antiviral activity against SARS-CoV-2. The nanomicelles, formulated using the direct dissolution method, exhibited an average size of 37.4 ± 8.01 nm and a polydispersity index (PDI) of 0.427 ± 0.01. Their antiviral efficacy was assessed in SARS-CoV-2-infected Vero E6 and Calu-3 cell models, where treatment with a 1:2 dilution inhibited viral replication by more than 90%. Cytotoxicity assays confirmed the nanomicelles were non-toxic to both cell lines after 72 h. In SARS-CoV-2-infected Calu-3 cells (human type II pneumocyte model), treatment with Pluronic F127-based nanomicelles containing atazanavir (ATV) significantly reduced viral replication, even under high MOI (2) and after 48 h, while also preventing IL-6 upregulation. To investigate their mechanism, viral pretreatment with nanomicelles showed no inhibitory effect. However, pre-exposure of Calu-3 cells led to significant viral replication reduction (>85% and >75% for 1:2 and 1:4 dilutions, respectively), as confirmed by transmission electron microscopy. These findings highlight Pluronic F127-based nanomicelles as a promising nanotechnology-driven strategy against SARS-CoV-2, reinforcing their potential for future antiviral therapies.

## 1. Introduction

Despite significant efforts by the scientific community, there is still no highly effective antiviral molecule for the treatment of moderate and severe cases of COVID-19. The available therapeutic strategies are limited in their efficacy, particularly in preventing viral replication in advanced stages of infection. In this context, nanotechnology has emerged as a powerful tool for the development of innovative antiviral strategies, providing nanostructured drug delivery systems capable of enhancing drug bioavailability, cellular uptake, and therapeutic effectiveness. Several recent studies have explored the potential of nanotechnology-based approaches, including polymeric nanocarriers, lipid-based formulations, and functionalized nanoparticles, to improve the targeting and controlled release of antiviral agents [1].

Nanomaterials have also demonstrated direct antiviral activity by interfering with viral entry, replication, and immune modulation. For instance, advanced delivery platforms utilizing nanocarriers, such as pegylated graphene oxide and carbon quantum dots, have shown promising antiviral effects against SARS-CoV-2, either through enhanced drug delivery or direct interaction with viral structures [2]. Additionally, the targeted functionalization of nanostructures has facilitated the development of efficient antiviral therapies with minimized toxicity, offering a promising avenue for future antiviral drug development [3]. These findings underscore the potential of nanotechnology as an innovative strategy for combating viral infections, particularly COVID-19, through both drug delivery enhancement and direct antiviral mechanisms.

Among the various nanosystems available, polymeric nanomicelles have gained attention as promising carriers for antiviral molecules due to their ability to improve solubility and stability while minimizing toxicity. These self-assembled nanostructures, composed of amphiphilic block copolymers, have demonstrated efficacy in increasing drug retention at target sites, potentially improving antiviral performance. Recent studies have explored the use of nanomicelles for the delivery of antiviral drugs, demonstrating their potential in combating viral infections, including those caused by coronaviruses [4,5].

One of the most studied amphiphilic copolymers for nanomicelle formation is Pluronic F127 (Poloxamer 407), a non-ionic triblock copolymer composed of polyethylene oxide (PEO) and polypropylene oxide (PPO) blocks. This surfactant is approved by the FDA for pharmaceutical applications and is widely used as an excipient in drug formulations due to its biocompatibility, low immunogenicity, and ability to enhance drug solubility [6,7,8]. Additionally, Pluronic F127 has been recognized as an adjuvant in vaccine formulations, contributing to enhanced immune responses [9,10]. Beyond its role as a formulation excipient, recent studies have suggested its potential antiviral properties, particularly against enveloped viruses. These findings suggest that Pluronic F127-based nanomicelles could serve a dual function: improving drug delivery while exerting direct antiviral effects [11,12].

Atazanavir (ATV), an antiretroviral protease inhibitor commonly used in HIV therapy, has been identified as a candidate for drug repurposing against SARS-CoV-2. In vitro and in silico studies have shown that ATV can bind to and inhibit the SARS-CoV-2 main protease (Mpro), a key enzyme for viral replication. It not only reduced viral titers in infected Vero and human pulmonary epithelial cells but also decreased pro-inflammatory cytokine production, such as IL-6 and TNF-α. More recently, in vivo studies using K18-hACE2 transgenic mice demonstrated that oral administration of ATV conferred partial protection against lethal SARS-CoV-2 challenge, reducing lung inflammation and cell death. These findings support the potential of ATV as an antiviral agent for COVID-19 and justify its incorporation into nanocarrier-based delivery systems for enhanced therapeutic outcomes [13,14].

SARS-CoV-2, the causative agent of COVID-19, belongs to the *Betacoronavirus* genus and primarily infects human alveolar epithelial cells, endothelial cells, and smooth muscle cells via the binding of its Spike (*S*) protein to the angiotensin-converting enzyme 2 (ACE-2) receptor. This interaction is crucial for viral entry, as it is followed by membrane fusion, viral genome release, and replication within the host cell [15,16,17]. Viral entry is further facilitated by host proteases, such as transmembrane serine protease 2 (TMPRSS2), which primes the *S* protein for fusion [16,17,18]. Once inside the cell, SARS-CoV-2 triggers an inflammatory cascade, often leading to a cytokine storm, characterized by excessive production of inflammatory mediators, including interleukin-6 (IL-6) and interleukin-8 (IL-8) [19,20].

Considering these mechanisms, polymeric nanomicelles, particularly those based on Pluronic F127, represent a potential strategy for inhibiting SARS-CoV-2 infection at different stages. These nanomicelles may disrupt viral interactions with host cells by interfering with membrane fusion, preventing viral entry, or modulating inflammatory responses. In this study, we developed Pluronic F127-based polymeric nanomicelles containing ATV and evaluated their antiviral potential against SARS-CoV-2. We assessed their capacity to reduce viral replication in SARS-CoV-2-infected Calu-3 cells (a model of human type II pneumocytes) and examined their effects on IL-6 and IL-8 production. Furthermore, we explored the potential mechanism of action by evaluating whether the nanomicelles act directly on viral particles or influence viral entry into host cells. Our findings suggest that Pluronic F127 nanomicelles may serve as an innovative nanotechnology-driven strategy for combating SARS-CoV-2 and potentially other emerging enveloped viral threats.

## 2. Materials and Methods

### 2.1. Poloxamer 407 Characterization and CMC Determination Methodology

In order to determine the critical micellar concentration of the poloxamer used in this paper, a methodology adapted from Samith et al. (2013) was used [21]. For this purpose, the dye solubilization method was performed, followed by reading using a spectrophotometer. Poloxamer 407 solutions in water varying from 0.001 to 6 mg/mL were produced, and the dye used to determine micelle formation was Sudan Black. Thus, 5 mg of Sudan was solubilized in 5 mL of Pluronic solution and subsequently analyzed in a spectrophotometer at 656 nm (maximum wavelength of the dye). This study was carried out at room temperature (25 °C), and the absorbance results obtained were used to create an absorbance versus poloxamer concentration graph.

### 2.2. Development of Nanomicelles

The polymeric micelles were developed using the direct dissolution method, following a methodology adapted from Feitosa et al. (2019), Sotoudegan et al. (2016), and Yang et al. (2009) [22,23,24]. For this purpose, poloxamer 407 was added to distilled water at a 10% (*w/v*) concentration. The solution was kept under stirring at 40 °C for 24 h until the polymer was completely solubilized. The micelles formed were later filtered through a 0.45 µm Millipore filter for subsequent analysis.

### 2.3. Size, Polydispersity Index Analysis (PDI), Zeta Potential (mV), and TEM (Transmission Electron Microscopy)

After preparing the nanomicelles, 1 mL aliquots were taken for mean size (diameter in nm) and Polydispersity Index (PDI) analysis. Dynamic light scattering was used with a Zetasizer Nano^®^ S90 device (Malvern Instruments^®^, Worcestershire, UK). The analyses were carried out in triplicate and the results were expressed as mean ± standard deviation.

The Nanomicelle solution at 10% (*w/w*) was added to the cell, and the zeta potential was measured using a Zetasizer Ultra device (Malvern Instruments^®^, Worcestershire, UK). Zeta potential (mV) was measured to estimate the charge of polymeric nanomicelles.

Nanomicelles solution was diluted 1:5 and added to a copper grid (300 mesh with Formvar coating—Electron Microscopy Sciences, Hatfield, PA, USA) and observed using a Hitachi HT 7800 transmission electron microscope (Hitachi, Chiyoda, Tokyo, Japan).

### 2.4. In Vitro Release Study of ATV

To evaluate the in vitro release profile of ATV encapsulated in polymeric nanomicelles, a diffusion cell containing a cellulose acetate membrane (molecular weight limit of 14,000 Daltons, Sigma-Aldrich, Burlington, MA, USA) was used to separate the donor and receptor compartments, a thermostatic bath for temperature control (37 °C), and a magnetic stirring plate for homogenization of the receptor medium. The receptor medium was composed of sodium phosphate buffer solution pH 7.4 with 0.2% sodium lauryl sulfate. Sodium lauryl sulfate was used to improve the solubility of ATV in the aqueous receptor medium. ATV encapsulated in polymeric nanomicelles at 0.5 µg/mL was placed in the donor compartment. Cellulose acetate membrane was used to separate the donor compartment from the receptor medium. This system was kept under magnetic stirring at a temperature of 37 °C and protected from light. At pre-established time intervals (1, 2, 3, 4, 5, 6, 24, 28, 30, 48, and 50 h), 2.5 mL aliquots were removed from the receptor medium and analyzed using a spectrophotometer (V-630, Jasco, Japan) at the maximum absorption wavelength of ATV (ʎ = 248 nm). After analysis, the aliquot of 2.5 mL was transferred to the receptor medium of the diffusion cell to continue the in vitro release study. The absorbances obtained were used to calculate the concentration of ATV released from the polymeric nanomicelles using the analytical curve. The concentrations obtained were used to construct the release profile graph. The experiments were carried out in quintuplicates (*n* = 5 determinations). After obtaining the in vitro release profile, two kinetic models were applied to the release study data to understand the parameters that control the release of the antiviral from the nanomicelles. The kinetic models were the “Zero Order Model” and the “Higuchi Model”. After applying the kinetic model and linearization, the coefficient of determination (R^2^) was obtained. The coefficients of determination were compared to choose the appropriate kinetic model [25,26]. Spectrophotometry was chosen to quantify ATV released from nanomicelles. The methodology used was adapted in studies carried out by Ghante et al. (2014) [27]. The drug solution was previously prepared in methanol at a concentration of 100 µg/mL. The methanolic solution was diluted in sodium phosphate buffer containing 0.2% sodium lauryl sulfate in the range of 0.6 to 6 µg/mL. To evaluate the linearity of the analytical curve, the dilutions were analyzed in a spectrophotometer using a wavelength of 248 nm. Sodium phosphate buffer pH 7.4 with 0.2% sodium lauryl sulfate was used as a blank to adjust the spectrophotometer. The selectivity of the method was evaluated by spectrophotometric analysis of: ATV-diluted methanol, ATV encapsulated in polymeric nanomicelle diluted in receptor medium from the in vitro release study, and polymeric nanomicelle (without antiviral) diluted in receptor medium from the in vitro release study.

### 2.5. Cell and Virus Culture

Vero E6 cells (African green monkey kidney cells, ATCC CRL-1586) and Calu-3 cells (type II human pneumocyte model, kindly provided by the Farmanguinhos RPT11M platform) were cultured in Dulbecco’s modified Eagle’s medium with high glucose content (DMEM; GIBCO, Waltham, MA, USA) supplemented with 10% fetal bovine serum (FBS; GIBCO, Brazil South America), 100 U/mL penicillin, and 100 µg/mL streptomycin (GIBCO, Waltham, MA, USA). The cells were grown in 96-well plates (Kasvi) (1.0 × 10^4^ Vero E6/well or 2.0 × 10^4^ Calu-3/well) and 6-well plates (4.0 × 10^5^ Calu-3/well) in an incubator at 37 °C with a 5% CO_2_ atmosphere. After 1 (Vero E6) or 4 (Calu-3) days, the cultures were processed for different assays. The SARS-CoV-2 isolate (strain B.1, GenBank #MT710714, SisGen AC58AE2) was stored at −80 °C and handled in a biosafety level 3 (BSL3) environment, in accordance with the recommendations of the World Health Organization [28].

### 2.6. Ultrastructural Analysis of Micelles and SARS-CoV-2 Particles by Negative Staining Technique

One drop of the suspended micelles or viral suspensions was applied onto a Formvar-covered electron microscope 400-mesh copper grid (Electron Microscopy Sciences, Hatfield, PA, USA). After 15 s, the excess sample was removed with filter paper, and a drop of 2% phosphotungstic acid (PTA), with a pH of 7.0, was applied. The excess of PTA was removed after 15 s, and the grid was examined by a Hitachi HT 7800 (Hitachi, Chiyoda, Tokyo, Japan) transmission electron microscope [29,30]. For biosafety level reasons, 1% glutaraldehyde diluted in sodium cacodylate buffer (1:1) was added to non-inactivated SARS-CoV-2 samples prior to grid preparation and transmission electron microscopy (TEM) analysis.

### 2.7. Cytotoxicity Test

Calu-3 and Vero E6 cells were treated with different proportions of the 1:X micelles (X = 2, 4, 8, 16, 32, 64, 128, 256, or 512). The cell viability of the culture was determined using a colorimetric assay detecting the activity of the enzyme Lactate Dehydrogenase (LDH) in the culture supernatant using the CytoTox^®^ Kit (Promega, Madison, WI, USA) in 96-well plates (Kasvi, Curitiba, Brazil), according to the manufacturer’s instructions. The manufacturer’s own 1x lysis solution was used as a positive control for the LDH release reaction. The reading was measured at 490 nm on the LMR 96 equipment (LMR-96i-4, Loccus, São Paulo, SP, Brazil).

### 2.8. Evaluation of the Micelles’ Activity on SARS-CoV-2 Replication and Cytokine Production

Calu-3 and Vero E6 cells were infected with SARS-CoV-2 at a multiplicity of infection (MOI) of 0.01 for 1 h at 37 °C with a 5% CO_2_ atmosphere. After this period, the supernatant was replaced by another containing different proportions of the 1:X micelles (X = 2, 4, 8, 16, 32, 64, 128, 256, or 512). After 24 h, the supernatant was collected, and the viruses grown in the presence or absence of micelles were titrated by plaque-forming units’ assay. Alternatively, Calu-3 cells were infected at a multiplicity of infection (MOI) of 2.0. After 1 h, the culture medium was replaced with fresh medium containing nanomicelles at 1:2 or 1:4 dilutions with or without ATV sulfate at concentrations of 250 µg/mL or 125 µg/mL, respectively. After 48 h of infection, the culture medium was collected for viral titration and cytokine quantification. For titration, the supernatant was diluted in culture medium in base 2 series (1:2 to 1:128). A volume of 50 µL/well of this dilution was added to Vero E6 cells, previously grown in 96-well plates (1 × 10^4^ cells/well; Kasvi) and maintained at 37 °C, 5% CO_2_. After 1 h, we added DMEM (10×) (GIBCO, Waltham, MA, USA) cell culture containing 2% fetal bovine serum (FBS; GIBCO, Brazil, South America) and 2.4% carboxymethylcellulose (Sigma-Aldrich, Burlington, MA, USA), and the cells were kept at 37 °C for 72 h. After this period, the cells were fixed with a 4% formalin solution for 3 h at room temperature. The cells were then stained with a 0.04% crystal violet solution for 1 h at room temperature. The cell culture was washed in water to remove the staining solution and, after drying, the Plaque Forming Units (PFU/mL) were quantified [31,32,33]. For the quantification of IL-6 and IL-8 cytokine production, the Human Standard ABTS ELISA Development kit (Peprotech, Thermo Fisher Scientific, Waltham, MA, USA) was used, following the manufacturer’s recommendations.

### 2.9. Evaluation of the Virucidal Activity of Micelles on SARS-CoV-2

The micelles were diluted in DMEM (GIBCO, Waltham, MA, USA) in the following proportions: 1:2 or 1:4, in a final volume of 250 µL. The SARS-CoV-2 viral isolate was then added to the micelle solution or to the culture medium alone (control) at a MOI equivalent to 0.01 (5.0 × 10^2^ PFU for 5 wells–quintuplicate), and incubated at room temperature for 5 or 30 min. After this period, viral titration was carried out with serial base 2 dilutions (1:2 to 1:128) of the solution (virus with micelle or control) in a final volume of 50 µL/well in Vero E6 cells, previously cultured in 96-well plates (1 × 10^4^ cells/well; Kasvi) and kept at 37 °C, 5% CO_2_ for 1 h. Subsequently, DMEM (10×) (GIBCO, Waltham, MA, USA) containing 2% fetal bovine serum (FBS; GIBCO, Brazil, South America) and 2.4% carboxymethylcellulose (Sigma-Aldrich, Burlington, MA, USA) was added to the cell culture, kept at 37 °C for 72 h, and the PFU titration protocol was followed.

### 2.10. Evaluation of the Micelle’s Activity on the SARS-CoV-2 Adsorption

The micelles were diluted in DMEM (GIBCO, Waltham, MA, USA) in proportions of 1:2 and 1:4, in a final volume of 500 µL. The solution was gently homogenized. The SARS-CoV-2 viral isolate was then added to the solution or to the culture medium alone (control) at a MOI equivalent to 0.01 (2.0 × 10^3^ PFU for 10 wells). Next, the solution (virus with micelle or control) in a final volume of 50 µL was added to the Calu-3 cells, previously cultured in a 96-well plate (2.0 × 10^4^ cells/well) and kept at 4 °C and 5% CO_2_ for 1 h. Subsequently, the culture supernatant was exchanged for new medium containing DMEM (GIBCO, Waltham, MA, USA) with 10% fetal bovine serum (SFB; GIBCO, Brazil, South America) in a final volume of 100 µL/well, and the culture was incubated at 37 °C and 5% CO_2_ for 24 h. After this period, the culture supernatant was collected for viral titration by plaque assay on Vero E6 cells.

### 2.11. Pre-Treatment Test with Micelles on SARS-CoV-2 Replication

The micelles were diluted in DMEM (GIBCO, Waltham, MA, USA) in proportions of 1:2, 1:4, and 1:8, in a final volume of 500 µL. The solution was gently homogenized. Next, the solution (micelle or control DMEM) in a final volume of 50 µL was added to the Calu-3 cells, previously grown in a 96-well plate (2.0 × 10^4^ cells/well) and maintained at 37 °C and 5% CO_2_. After 1 h, the medium was replaced with 50 µL of another medium containing the SARS-CoV-2 viral isolate at a MOI of 0.01 (2.0 × 102 PFU/well), and the cells were kept at 4 °C and 5% CO_2_ for 1 h. The culture supernatant was then replaced with fresh medium containing DMEM with 10% FBS (GIBCO, Brazil, South America) in a final volume of 100 µL/well, and the culture was kept at 37 °C and 5% CO_2_ for 24 h. After this period, the culture supernatant was collected for viral titration by plaque assay on Vero E6 cells.

### 2.12. Biological Assays to Evaluate SARS-CoV-2 Replication in Calu-3 Cells by TEM

Calu-3 cells were grown in 6-well plates at a density of 4.0 × 10^5^ cells/well. First, the cells were infected and then treated at a 1:2 dilution, as already described in the assay to evaluate the micelles on SARS-CoV-2 replication. After 48 h, the monolayer was washed with PBS 1x, the cells were loosened with 500 µL of 0.25% trypsin, and then 500 µL of FBS (GIBCO, Brazil, South America) was added to inactivate the trypsin. This solution was diluted 1:2 in 2.5% glutaraldehyde buffer and then prepared for TEM. In another moment, the pre-treatment assay was repeated in 6-well plates with a 1:2 micelle ratio, followed by infection with an MOI of 3. In this case, the monolayer was washed and collected, as described above, immediately after the cells had been infected at 4 °C and 5% CO_2_ for 1 h, and then the monolayers were processed for TEM analysis.

### 2.13. Processing of Sample Cells for Ultrastructural Analysis by TEM

Cell suspensions were fixed in 2.5% glutaraldehyde in sodium cacodylate buffer (0.2 M, pH 7.2) and postfixed in 1% buffered osmium tetroxide, dehydrated in acetone, embedded in epoxy resin, and polymerized at 60 °C over 3 days [30,34]. Ultrathin sections (50–70 nm) were obtained from the resin blocks. The sections were picked up using copper grids (300 mesh and no coating–Electron Microscopy Sciences, Hatfield, PA, USA) and observed using a Hitachi HT 7800 (Hitachi, Chiyoda, Tokyo, Japan) transmission electron microscope.

### 2.14. Statistical Analysis

Statistical analyses were conducted using one-way ANOVA, followed by either Tukey’s or Dunnett’s multiple comparison tests, as appropriate. Data are presented as the mean ± standard deviation (SD) from three to five independent experiments. A *p*-value of ≤0.05 was considered statistically significant. Graphs were generated using GraphPad Prism version 10.2.1 (GraphPad Software, La Jolla, CA, USA) for Windows.

## 3. Results and Discussion

### 3.1. Poloxamer 407 Characterization and CMC Determination

Poloxamers are widely used in the pharmaceutical area due to their various properties, such as stabilizers, emulsifiers, and solubilizers [10,35,36,37,38]. Use of these polymers in the development of nanosystems will depend mainly on their characteristics related to micelle formation for drug encapsulation. For the micellization process to occur, these substances must be solubilized in an aqueous medium above the critical micellar concentration, that is, the concentration at which micelles begin to form [39].

Various techniques can be used to determine the CMC of the poloxamers. Solubilization of a dye in copolymer solutions followed by ultraviolet-visible analysis is a widely used method. Below the CMC, the solubility of the dye is low due to its lipophilicity. As the poloxamer concentration increases until reaching the CMC, the solubility of the dye increases with its incorporation into the micelles formed [39,40].

The critical micellar concentration of poloxamers can vary depending on the method chosen for determination. Thus, various values can be found for the CMC of poloxamer 407, ranging from 0.001 to 10 mg/mL [21,39,41,42]. In this paper, the poloxamer’s CMC was estimated at 25 °C using the method of solubilizing the Sudan dye in solutions with different concentrations of the surfactant, followed by ultraviolet-visible analysis. This dye has lipophilic characteristics; therefore, at concentrations below the CMC, low absorbance is detected in the ultraviolet due to the low solubility of the dye in aqueous media [43].

**Figure 1:**
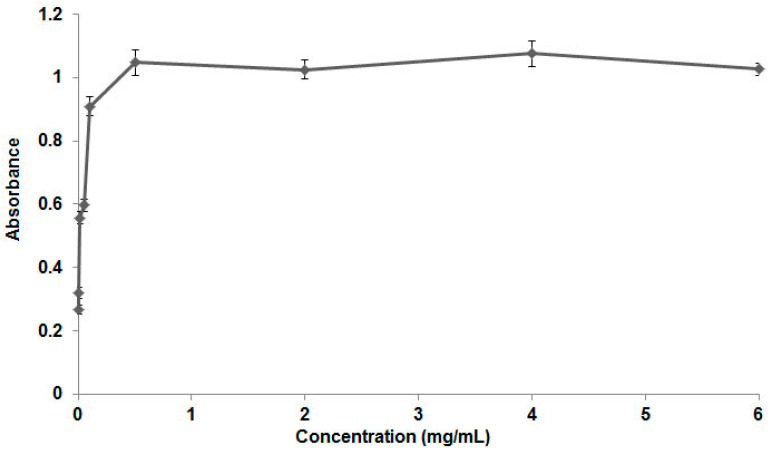
Graph for determining poloxamer 407′s critical micellar concentration. As can be seen in the absorbance versus concentration graph (mg/mL), the CMC of poloxamer varied between 0.1 and 0.5 mg/mL. The absorption wavelength used to determine the CMC was 656 nm.

As can be seen in the graph in Figure 1, the CMC of the poloxamer varied between 0.1 and 0.5 mg/mL, which is in line with the values found in the literature. As the poloxamer concentration increases, micelles begin to form, the dye is incorporated and solubilized in the surfactant solution, and the absorbance value increases. The maximum point on the curve represents the CMC of the poloxamer.

### 3.2. Development of the Nanomicelles, Size, Polydispersity Index, and Zeta Potential

Polymeric micelles are systems characterized by simple preparation techniques and easier scaling. The direct dissolution technique used in this paper to prepare the micelles is characterized by its simplicity and has the advantage of not requiring organic solvents [36,43]. This method can only be used for water-soluble surfactants that have spontaneously formed micelles, as is the case with poloxamer 407 [36,44,45].

Poloxamer 407 was chosen for preparing the nanomicelles because of its characteristics, such as biocompatibility and low toxicity risk. When solubilized in an aqueous medium, this polymer undergoes a spontaneous micellization process. The outer portion of the micelles is made up of polyethylene glycol, a substance widely used in the production of nanosystems for transport purposes in biological media due to its high hydration and mobility in aqueous media, which hinders interaction with the endothelial system and reduces the elimination by excretion processes [36,46,47,48]. The nanomicelles were prepared using the direct dissolution method, which has the advantage of not requiring organic solvents.

The mean size and distribution of the particles in solution are parameters highly evaluated during the development of nanosystems because they provide information on stability, formation of aggregates in the dispersion, and particle size [49,50]. These characteristics will determine the behavior of these systems after in vivo administration, such as pharmacokinetic parameters [51,52]. In this paper, nanomicelles with a size of 37.4 ± 8.01 nm and a PDI of 0.427 ± 0.01 were developed. The size of the nanosystems will influence distribution and bioavailability after administration. Particles larger than 100 nm can present a lower distribution because they are more easily phagocytosed by the mononuclear phagocytic system. This will also depend on the material used to prepare the systems [53].

The methods employed to prepare polymeric micelles are characterized by producing systems on the nanometric scale with sizes between 10 and 100 nm, depending on the size of the hydrophobic and hydrophilic segments and on the molecular weight of the polymers used. Micelles between 10 and 70 nm in size are more advantageous due to their ability to escape elimination processes, in addition to possessing greater efficiency in tissue distribution and longer circulation time. Particles between 5 and 10 nm are rapidly eliminated by renal excretion, and those above 200 nm are easily removed by the reticuloendothelial system cells [36,45,54]. Hydrophilic blocks can be added to the copolymer structures, conferring escape properties. Neutral and hydrophilic surfaces increase circulation time, whereas positively charged surfaces are less stable due to aggregation between the micelles [43,54,55].

The literature already describes polymeric micelles produced from poloxamer 407 with sizes of less than 50 nm. In relation to the polydispersity index, it is known that the hydrophilic portions of polymers tend to group together in aqueous solutions, which leads to the formation of thermodynamically reversible aggregates. For this reason, micelles are highly polydisperse [23,56,57].

The zeta potential of the nanomicelle solution was measured in triplicate, and the value obtained was −1.503 ± 0.176 mV. The micelle has a small negative charge value and, consequently, is close to the neutral charge of 0 mV. The zeta potential determination graph is shown in Figure S1 (Supplementary Materials). This result is expected because nanomicelles are formed by Pluronic F127, which is a non-ionic polymer.

Nanomicelles have a small negative charge with the possibility of electrostatic interaction with SARS-CoV-2. The charge of the spike protein is responsible for the interactions of the virus with the environment and can change between variants. Wild Type (WT) has a negative surface charge, the Delta variant has a neutral charge, and Omicron has a positive charge [58]. The positively charged variants have a greater possibility of electrostatic interaction with negatively charged nanomicelles of Pluronic F127. However, nanomicelles have a small negative charge and are close to the neutral charge of 0 mV; thus, this nanosystem is capable of interacting with negatively charged and neutral variants.

### 3.3. Morphological Characterization of Nanomicelles and SARS-CoV-2 Particles by TEM

**Figure 2:**
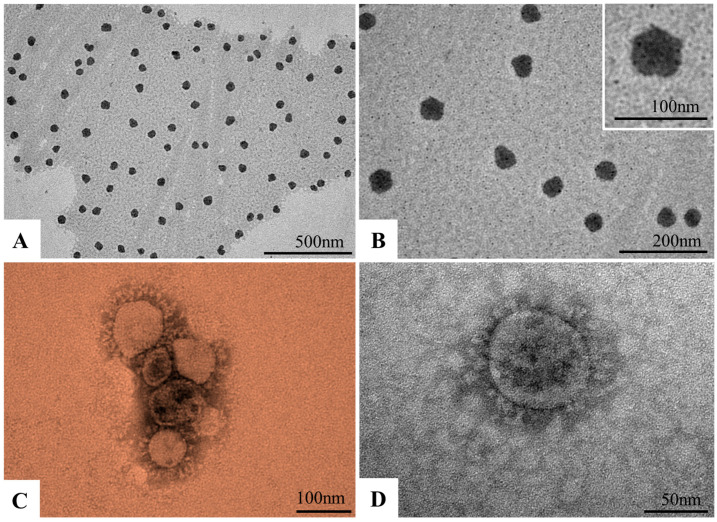
Morphological characterization of micelles and SARS-CoV-2 particles by negative staining technique. Analyses of the micelles (**A**,**B**) showed structures with a spherical morphology and with a mean diameter of 50 nanometers. The SARS-CoV-2 particles showed a spherical morphology exhibiting projections on the envelope (spike protein) with a mean diameter of 76 nanometers (**C**,**D**). (**C**) has been colored in Adobe Photoshop.

Ultrastructural analysis of nanomicelles showed electron-dense structures with a spherical morphology and with a mean diameter of 50 nanometers (Figure 2A,B). The SARS-CoV-2 particles showed a spherical morphology, exhibiting projections on the envelope (spike protein) with a mean diameter of 76 nanometers (Figure 2C,D), which is characteristic of viruses belonging to the *Coronaviridae* family.

### 3.4. The Micelles Were Not Toxic to the Main Cell Model Studied and Were Able to Inhibit the Replication of SARS-CoV-2

**Figure 3:**
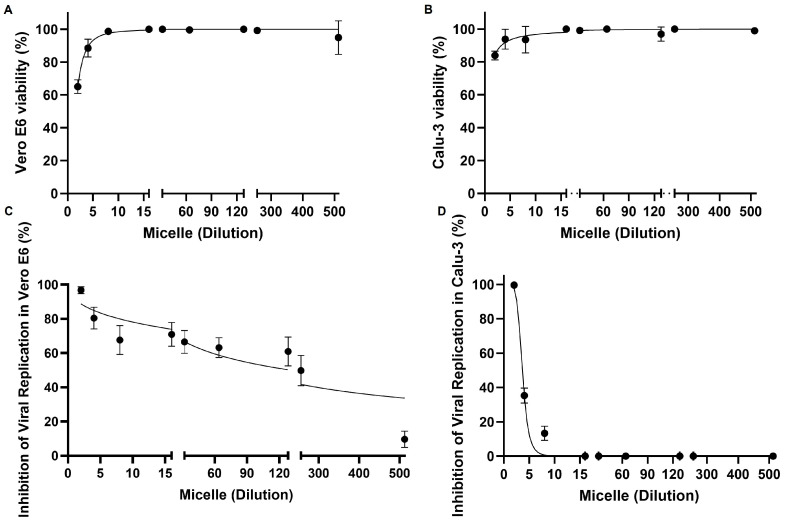
Viability of Vero E6 and Calu-3 cells in the presence of micelles and micelles inhibition of SARS-CoV-2 replication with different magnitudes in the cell models tested. (**A**) Vero E6 cells (1 × 10^4^ cells/well) or (**B**) Calu-3 cells (2 × 10^4^ cells/well) were exposed to micelles in different 1:X ratios (X = 2, 4, 8, 16, 32, 64, 128, 256, or 512) and kept at 37 °C, 5% CO2 for 72 h. The viability of Vero E6 and Calu-3 cells was assessed by LDH dosage; the positive control of the assay was lysed cells (1× lysis solution). (**C**) Vero E6 cells (1 × 10^4^ cells/well) or (**D**) Calu-3 cells (2 × 10^4^ cells/well) were infected with SARS-CoV-2 (MOI: 0.01) for 1 h at 37 °C and 5% CO_2_. The supernatant was then removed, and the cells were exposed to the micelles in different 1:X ratios (X = 2, 4, 8, 16, 32, 64, 128, 256, or 512) and kept at 37 °C, 5% CO_2_ for 24 h. Viral titer was determined by plaque assay. The x-axis refers to the dilution factor applied in the experimental setup. Data are represented as mean ± SD of 6 experiments, and graphs were created using GraphPad Prism 10.1.1 software.

To assess the potential cytotoxicity of the micelles, we performed a lactate dehydrogenase (LDH) release assay on the culture supernatant of Vero E6 (Figure 3A) and Calu-3 (Figure 3B) cells. The results indicated that the micelles were not toxic to either cell model after 72 h of treatment, with cell viability exceeding 90% at dilutions of 1:4 and higher (Figure 3). At a 1:2 ratio, the viability of Vero E6 cells was more affected than that of Calu-3 cells, though it remained above 80%. These findings confirm the biocompatibility of the micellar system, consistent with previous studies showing that Pluronic F127-based nanocarriers exhibit low cytotoxicity and high stability in biological systems [59].

Treatment with micelles after viral infection in Vero E6 resulted in over 50% inhibition of SARS-CoV-2 replication, except at the highest dilution tested (1:512) (Figure 3C). In Calu-3 cells, significant viral inhibition was observed at the lowest dilutions (1:2, 1:4, and 1:8) after 24 h of infection (Figure 3D). These results align with previous studies highlighting the antiviral potential of polymeric micelles. For instance, Pluronic F127-based nanomicelles have demonstrated the ability to enhance the antipathogenic efficacy of hydrophobic molecules by improving their cellular uptake and stability [60]. Moreover, surfactant-based nanocarriers have been reported to interfere with viral envelope integrity, potentially limiting viral entry into host cells [61].

**Figure 4:**
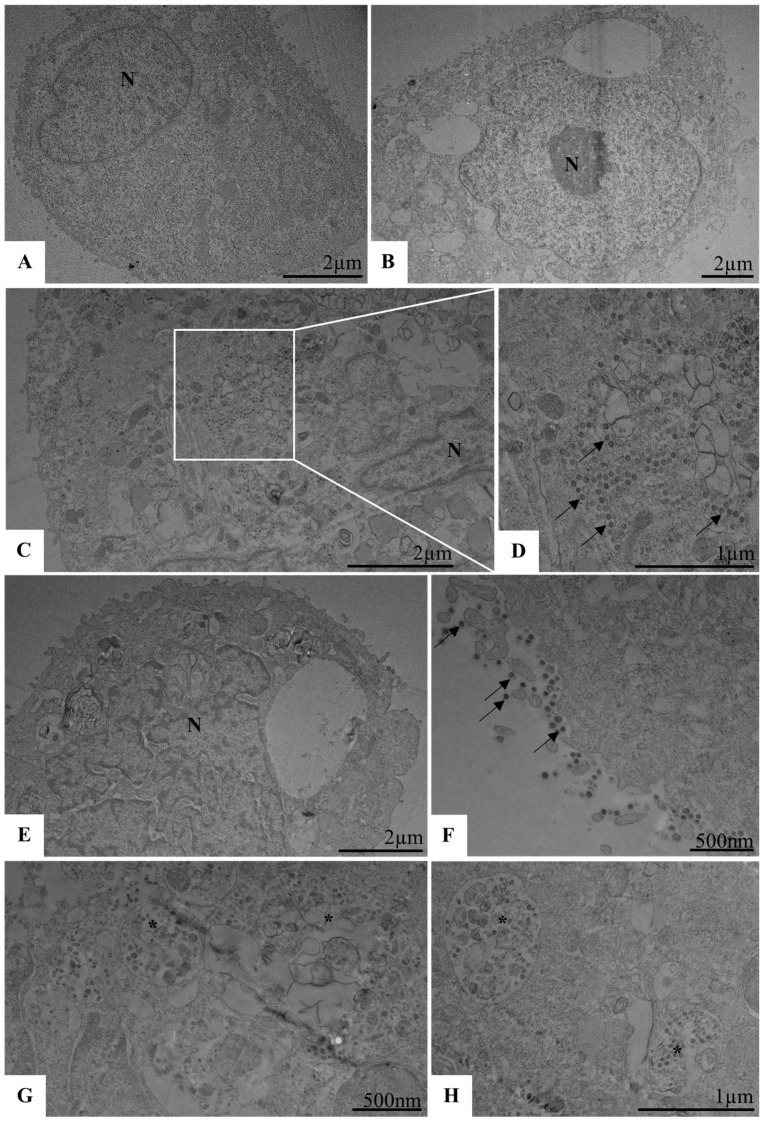
Ultrastructural analysis of Calu-3 cells infected with SARS-CoV-2 (MOI 0.01) and treated with micelle (1:2). (**A**,**B**) uninfected and untreated Calu-3 cells (control cell). (**C**,**D**) Calu-3 cells infected with SARS-CoV-2 (virus control); note the presence of SARS-CoV-2 particles inside the cytosol (arrows). (**E**) Calu-3 cell treated with micelle (micelle control). (**F**–**H**) Calu-3 cells infected with SARS-CoV-2 and exposed to the micelle; note the presence of defective SARS-CoV-2 particles in the lumen of cytosol vesicles (asterisk). N (Nucleus), SARS-CoV-2 particles (arrows).

We further confirmed the inhibitory effect of the micelles using transmission electron microscopy (TEM). Calu-3 cells seeded in 6-well plates were infected with SARS-CoV-2 (MOI 0.01) and exposed to micelles at a 1:2 dilution following the described protocol. After 48 h of treatment, cells were collected and processed for TEM imaging. No morphological alterations were observed in uninfected control cells or in cells treated with micelles alone (Figure 4A,B,E). Infected cells (infection control) exhibited SARS-CoV-2 particles within the cytoplasm (Figure 4C,D). However, in micelle-treated cells, viral particles were observed adhering to the cytoplasmic membrane, along with defective vesicles in the cytosol lumen (Figure 4F–H).

The Calu-3 cell model is particularly relevant for studying SARS-CoV-2, as it expresses both ACE-2 and TMPRSS2, two key factors for viral entry via membrane fusion [62,63]. Compared to Vero E6 cells, which rely primarily on endosomal entry mechanisms, Calu-3 cells provide a more physiologically relevant model for evaluating antiviral compounds [64,65,66]. The pronounced inhibition observed in Calu-3 cells suggests that the micelles may interfere with viral entry or membrane fusion, in line with previous findings demonstrating that surfactant-based formulations can alter lipid bilayer properties and impact viral-host interactions [67].

### 3.5. The Micelles Do Not Seem to Have Virucidal nor Anti-Adsorption Activity Against SARS-CoV-2

**Figure 5:**
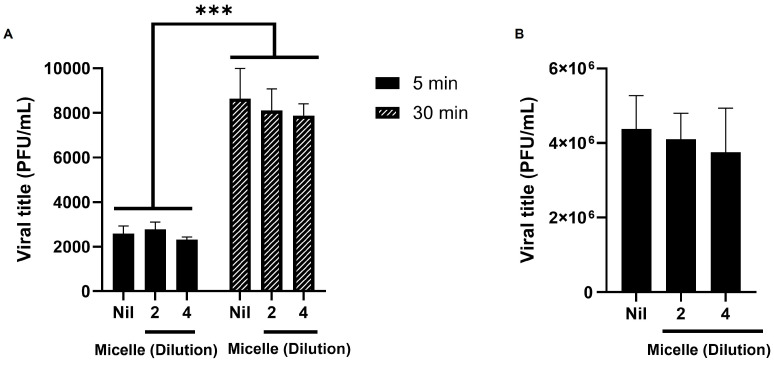
Micelles do not exhibit virucidal effects nor seem to affect SARS-CoV-2 adsorption to the host cell. (**A**) SARS-CoV-2 was exposed to different proportions (1:2 or 1:4) of micelle for 5 and 30 min. After this period, viral infectivity was evaluated in Vero V6 cells. Statistical analysis of SARS-CoV-2 titers in PFU/mL was carried out in comparison with the unexposed virus control (Nil) and pre-exposed virus with different dilutions in two different exposure times. (**B**) Calu-3 were infected and treated with SARS-CoV-2 and micelles (1:2 or 1:4) at 4 °C. After 1 h, the culture supernatant was exchanged for new medium containing DMEM with 10% fetal bovine serum, and the culture was incubated at 37 °C and 5% CO_2_ for 24 h; then the culture supernatant was collected for viral titration by plaque assay on Vero E6 cells. The x-axis refers to the dilution factor applied in the experimental setup. Data are represented as mean ± SD of 6 experiments, and graphs were created using GraphPad Prism 10.1.1 software, analyzed by one-way ANOVA followed by Tukey’s post-test (*n* = 6), *** *p* < 0.001.

We investigated whether the micelles could interfere with the early infection’s steps of SARS-CoV-2 replication. To this end, the micelles were diluted in different proportions (1:2 or 1:4) and then received viral particles. After 5 and 30 min of interaction, viral titration was performed in Vero E6 cells. The results showed that the pre-exposure of the virus with the micelles did not interfere with the viral infective capacity, since the viral titration of the pre-exposed virus did not differ significantly from the unexposed virus control (Nil) (Figure 5A).

We then further investigated whether the micelles could influence viral adsorption to the host cell. To this end, we infected and treated Calu-3 cells for 1 h at 4 °C, then replaced the culture medium with fresh medium containing 10% FBS and collected the supernatant after 24 h at 37 °C for viral titration in Vero E6 cells. At 4 °C, membrane fluidity is reduced, allowing viral particles to absorb to the cell surface but preventing penetration [68,69,70,71]. Thus, when the culture medium was replaced and the temperature was increased, only the particles that were successfully adsorbed were able to proceed with their replication cycle. In this assay, we also did not observe a direct effect of the micelles on SARS-CoV-2 replication (Figure 5B).

### 3.6. Pre-Treatment with Micelles Inhibits Viral Replication

**Figure 6:**
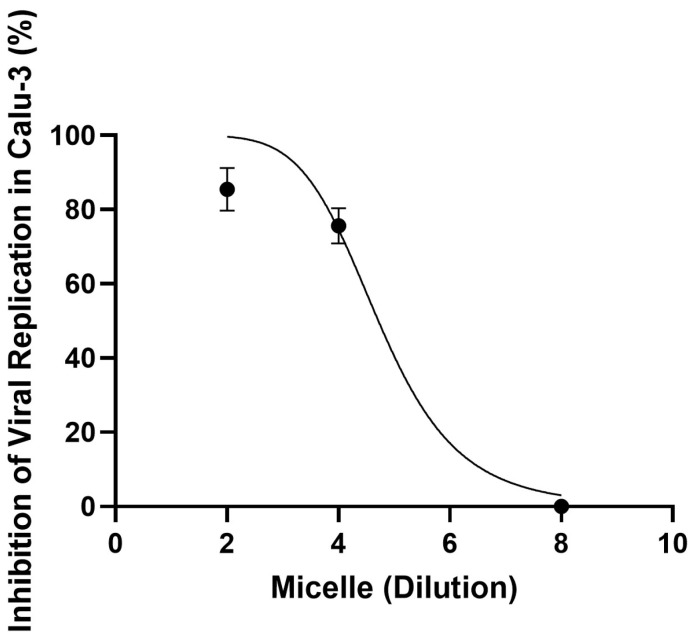
Pre-treatment of cells with the micelles inhibited SARS-CoV-2 replication. Calu-3 cells (2 × 10^4^ cells/well) were treated with the micelles in different proportions (1:2, 1:4, or 1:8) at 37 °C, 5% CO_2_ for 1 h. The medium was then changed to one containing only SARS-CoV-2 (MOI: 0.01), and the cells were incubated at 4 °C for 1 h. After this period, the medium was changed to one containing only 10% SFB. After 24 h, the supernatant was collected, and the viruses were titrated by plaque assay. The x-axis refers to the dilution factor applied in the experimental setup. Data represent the mean ± SD (*n* = 8).

To assess whether exposure to micelles could interfere with cell infectivity, Calu-3 cells were exposed to different dilutions of micelles (1:2, 1:4, or 1:8) for 1 h at 37 °C and 5% CO_2_. After this period, the medium was changed to one containing SARS-CoV-2, and the cells were placed at 4 °C for 1 h for virus adsorption. After this period, the culture medium was replaced with another containing only 10% FBS for 24 h, after which the supernatants were collected for virus titration in Vero E6 cells, following the previously described protocol. The data showed that pre-treatment with the micelles at dilutions of 1:2 and 1:4 inhibited the interaction of the virus with the cells; however, at a ratio of 1:8, no such inhibitory effect was observed (Figure 6).

**Figure 7:**
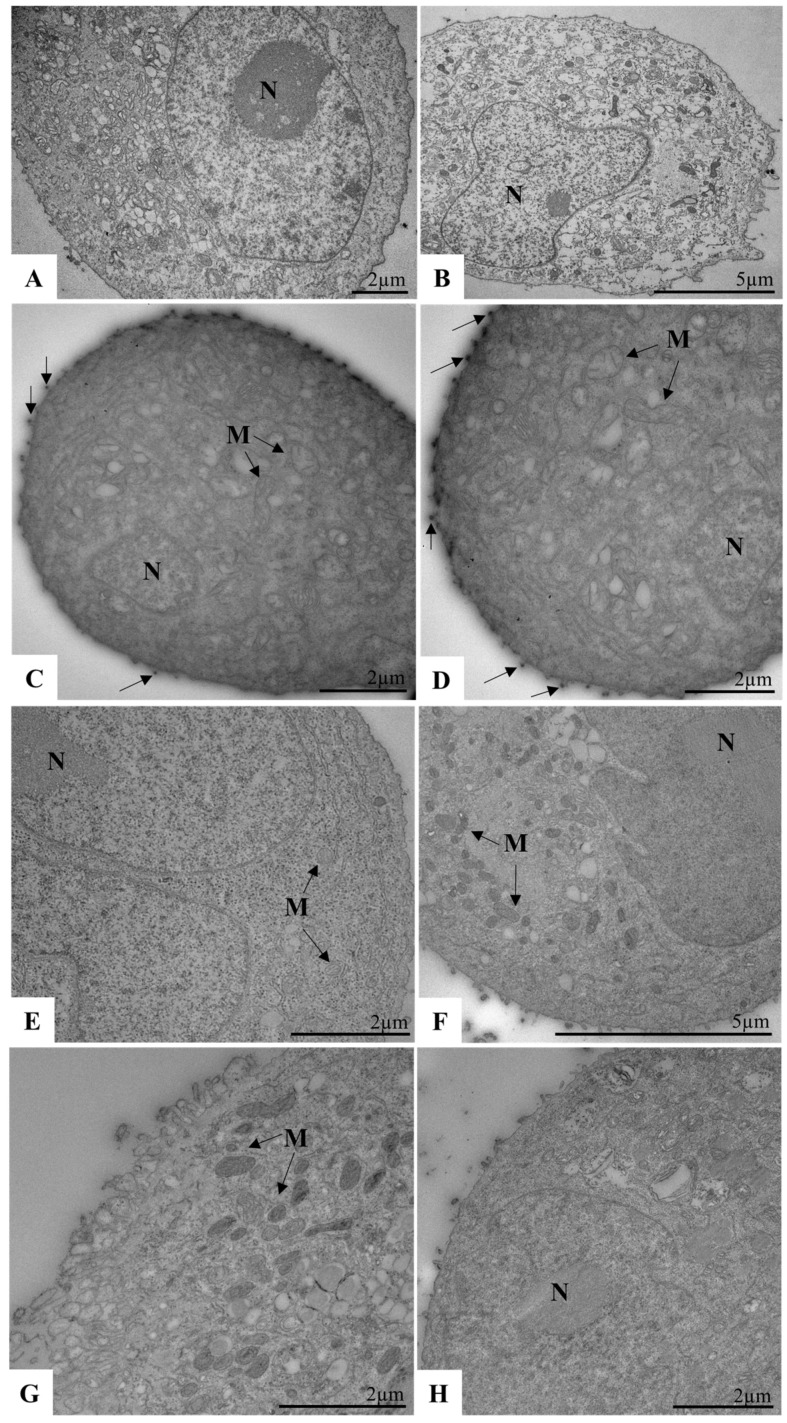
Ultrastructural analysis of Calu-3 cells pre-exposed to micelle (1:2) and infected with SARS-CoV-2 (MOI 3). (**A**,**B**) uninfected and untreated Calu-3 cells (control cell). (**C**,**D**) Calu-3 cells 1 hpi with SARS-CoV-2 (infected cell control); note SARS-CoV-2 particles on the cell membrane (arrow). (**E**,**F**) Calu-3 cells pre-exposed to micelle (micelle cell control). (**G**,**H**) Calu-3 cells pre-exposed to micelle and infected with SARS-CoV-2. N (Nucleus), M (Mitochondria).

We recorded the inhibitory effect using TEM. For this, Calu-3 cells in 6-well plates were exposed to the micelles in a 1:2 ratio, under the same conditions as above, and then infected with SARS-CoV-2 (MOI 3) for 1 h at 4 °C. After this period, the monolayer was collected for TEM processing. We observed no morphological changes in the control cells without pre-exposure to micelles or infection (Figure 7A,B), as well as in the cells pre-exposed to micelles (Figure 7E,F). As expected, we observed that in the infected control, the viruses are adsorbed to the cytoplasmic membrane (Figure 7C,D). However, in the cells pre-exposed to the micelles, this fact was not observed (Figure 7G,H). Then, the micelles were able to inhibit the interaction of the virus with the host cell before cell pre-exposition.

Several studies have highlighted the importance of viral adsorption as a key step in SARS-CoV-2 infection. The ability of the virus to attach to the host cell membrane depends on multiple factors, including membrane fluidity and the availability of ACE2 receptors [72]. It is well established that at 4 °C, viral particles can bind to the cell surface but cannot proceed to internalization due to reduced membrane fluidity [69]. Our findings align with these observations, as we observed viral adsorption in control conditions but not in cells pre-exposed to micelles.

A possible mechanism for this inhibition may involve alterations in the biophysical properties of the cell membrane. Studies on lipid-based formulations have demonstrated their ability to disrupt viral entry by modifying membrane microdomains, such as lipid rafts, which are crucial for viral attachment [73,74]. Given that SARS-CoV uses lipid rafts to enhance ACE2 receptor clustering and promotes efficient viral entry, it is plausible that micelles interfere with this process by affecting lipid organization at the plasma membrane [75]. Similar inhibitory effects have been observed with other amphiphilic molecules, such as cyclodextrins, which can alter lipid raft integrity and thereby prevent viral entry [76,77].

Furthermore, electron microscopy analysis provided direct evidence of this inhibitory effect, as viral particles were observed bound to the membrane in control cells but were absent in the micelle-treated cells. These results support the hypothesis that micelles disrupt viral adsorption, likely by modulating the physicochemical environment of the plasma membrane, an effect that has been proposed in previous studies for other lipid-based formulations [78].

### 3.7. Unveiling the Antiviral and Immunomodulatory Properties of Pluronic Micelles in SARS-CoV-2 Infection

While the antiviral potential of micelles is promising, their significance extends beyond their intrinsic activity. One of the most notable advantages of micelles is their ability to function as efficient drug delivery systems, enhancing the bioavailability and therapeutic efficacy of encapsulated molecules. Polymeric micelles, in particular, have gained attention for their role in improving drug solubility, stability, and targeted delivery to infected cells [79]. This property makes them attractive candidates for antiviral therapy, as they can be loaded with antiviral agents to potentiate their effects.

Several studies have demonstrated the effectiveness of polymeric micelles in delivering antiviral compounds. For example, polyethylene glycol (PEG)-based micelles have been successfully used to encapsulate hydrophobic antiviral drugs, improving their pharmacokinetics and therapeutic action against cancer and viruses, such as HIV [80,81,82,83]. Additionally, polymeric nanoparticles functionalized with targeting ligands have shown enhanced delivery to cancer cells and virus-infected cells, increasing drug accumulation and reducing systemic toxicity [84].

Lipid-based and polymeric micelle formulations, including those with pluronic, have been explored for the encapsulation of antiviral molecules to enhance their delivery to tissues and improve treatment outcomes [85,86,87,88]. The potential of micelles to disrupt viral entry mechanisms, combined with their ability to carry antiviral agents, represents a dual approach that could be explored further in the fight against COVID-19 and other viral infections.

Given these advantages, we next sought to evaluate whether micelles could act not only as direct inhibitors of viral adsorption but also as efficient carriers for antiviral compounds, further expanding their potential as therapeutic tools. For this purpose, infected Calu-3 cells (MOI 2.0) were exposed to micelles at 1:2 or 1:4 dilutions with or without ATV at concentrations of 250 µg/mL or 125 µg/mL, respectively. After 48 h of infection, the culture medium was collected for viral titration and cytokine quantification.

**Figure 8:**
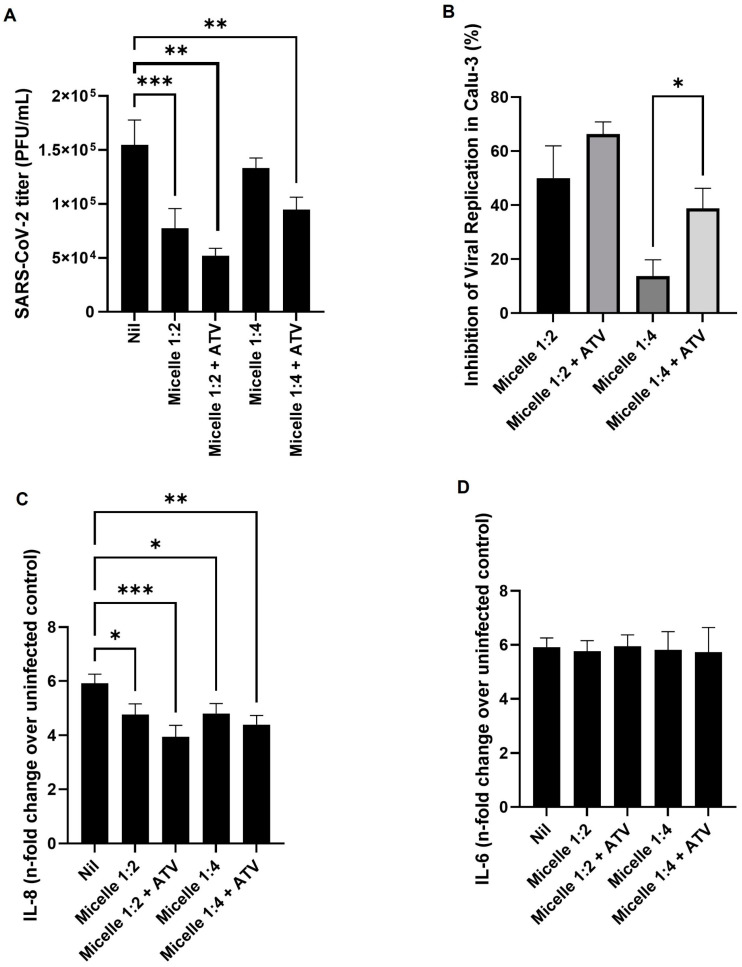
Calu-3 cells (6-well plates with 4.0 × 10^5^ cells/well) were infected with SARS-CoV-2 (MOI: 2) for 1 h at 37 °C and 5% CO_2_. The supernatant was then removed, and the cells were exposed to fresh medium containing nanomicelles at 1:2 or 1:4 dilutions with or without atazanavir (ATV) at concentrations of 250 µg/mL or 125 µg/mL, respectively, and kept at 37 °C, 5% CO_2_ for 48 h. (**A**) Viral titer was determined by plaque assay. (**B**) The inhibition percentage was also determined. (**C**) IL-6 and (**D**) IL-8 cytokine production was detected using a Human Standard ABTS ELISA Development kit (Peprotech, Thermo Fisher Scientific). Data are represented as mean ± SD of 6 experiments, and graphs were created using GraphPad Prism 10.1.1 software, analyzed by one-way ANOVA followed by Tukey’s post-test (*n* = 6), * *p* < 0.05, ** *p* < 0.01, *** *p* < 0.001.

We observed a statistically significant difference between the groups treated with micelles and the viral infection control (Figure 8A). Furthermore, the 1:2 micelle dilution was able to maintain its inhibitory potential against viral replication even when exposed to an MOI 200 times higher than initially tested and after 48 h of infection. The incorporation of ATV into the micelles further enhanced their inhibitory potential, showing a statistically significant difference when comparing the 1:4 micelle dilution with and without ATV (Figure 8B).

The excessive immune response known as a “cytokine storm” is a key driver of severe COVID-19 cases, often leading to systemic inflammation, multi-organ failure, and increased mortality [89,90]. Controlling this hyperinflammatory state is crucial to improving patient outcomes, as elevated levels of cytokines such as interleukin-6 (IL-6) are strongly associated with disease severity [91,92]. Various antiviral and immunomodulatory molecules have been investigated for their ability to mitigate the cytokine storm in SARS-CoV-2 infection. For instance, corticosteroids such as dexamethasone have demonstrated efficacy in reducing mortality by dampening excessive immune activation [93]. Additionally, other anti-inflammatory agents, such as baricitinib (a Janus kinase inhibitor) and tocilizumab (an IL-6 receptor antagonist), have been explored for their ability to suppress hyperinflammatory responses in severe COVID-19 cases [94,95,96].

Among antiviral agents with immunomodulatory properties, ATV has emerged as a promising candidate. ATV, a protease inhibitor traditionally used in HIV treatment, has been shown to exert anti-inflammatory effects by modulating cytokine production, including downregulation of IL-6 levels [13].

In severe cases of COVID-19, serum IL-6 levels show a significant increase, whereas IL-8 is more consistently detectable in patients with milder symptoms. Additionally, IL-8 levels have demonstrated a stronger correlation with overall clinical disease scores across different stages of infection compared to IL-6, indicating that these cytokines may play distinct roles in disease progression. Therefore, IL-6 and IL-8 could serve as valuable biomarkers for distinguishing severe COVID-19 cases and predicting disease outcomes [91]. Supporting this, a study by Alosaimi et al. (2021) further demonstrated that IL-6 and IL-8 levels were significantly elevated in critically ill COVID-19 patients who did not survive compared to those who recovered, highlighting their strong association with disease severity and mortality. Additionally, both cytokines correlated positively with in-hospital death, reinforcing their prognostic value. These findings suggest that while IL-6 is a key marker of severe inflammation, IL-8 may play an equally critical role in tracking disease progression, particularly in distinguishing mild from severe cases and predicting clinical outcomes [97].

Pluronics (also called Poloxamers), a class of amphiphilic block copolymers, have gained significant attention in biomedical research due to their unique ability to interact with cellular membranes [98,99]. Among them, Pluronic F127 has been widely studied for its potential as a drug delivery system, enhancing the solubility, stability, and bioavailability of therapeutic compounds. Its micellar structure allows for the encapsulation of hydrophobic drugs, protecting them from degradation and improving their targeted release at the site of action. This property has been particularly valuable in antiviral therapies, where controlled and localized drug release is crucial for effective viral suppression while minimizing systemic toxicity [100]. Recent studies have demonstrated that Pluronic F127-based micelles can enhance the pulmonary delivery of antiviral agents, such as remdesivir, a drug used in the treatment of SARS-CoV-2 infections. By incorporating remdesivir into Pluronic-based nanocarriers, researchers have achieved improved aerosolization, reduced cytotoxicity, and sustained drug release, making it a promising strategy for respiratory viral infections [100]. Although Pluronic F127 has been extensively explored as a carrier for antiviral drugs, no previous studies have reported an intrinsic antiviral activity of this polymer. Most of the research has focused on its role in improving drug solubility, enhancing cellular uptake, and modulating pharmacokinetics, but not on its potential direct effects against viral replication. This raises an intriguing question regarding whether Pluronic-based micelles might exert direct antiviral effects, independent of their drug-loading capabilities.

In this context, we evaluated the antiviral and immunomodulatory effects of micelles in the context of SARS-CoV-2 infection. Our results demonstrated that micelles at 1:2 and 1:4 dilutions, both with and without ATV, significantly inhibited SARS-CoV-2 replication and reduced IL-8 levels (Figure 8A–C). However, under the tested experimental conditions, no significant effects were observed on IL-6 levels (Figure 8D), suggesting that while these formulations can modulate IL-8-mediated inflammatory responses, they may not directly influence IL-6-driven pathways. These findings highlight the potential of micellar systems not only as antiviral carriers but also as immune-modulating platforms, warranting further investigation into their mechanisms of action and therapeutic applications. Given the role of IL-8 in COVID-19 progression, pluronic-based micellar formulations may serve as both immune response modulators and drug carriers, offering a dual approach to therapeutic intervention.

### 3.8. Spectrophotometric Validation, Release Studies, and Kinetic Modeling of Nanomicelles with ATV

Before starting the in vitro release study, the spectrophotometric method was validated. The maximum absorption of the antiviral in sodium phosphate buffer solution containing a solubilizing agent was 248 nm, which corroborates the wavelength for ATV found in the literature [27]. The polymeric nanomicelle solution without the antiviral showed small absorption at 190 nm. Therefore, there are no interferences for the quantification of ATV (λ = 248 nm) in the presence of polymeric nanomicelles (Supplementary Materials). With spectrophotometric analyses of ATV dilutions, an analytical curve was obtained with a coefficient of determination (R^2^) greater than 0.99, which confirms the linearity of the method as shown in Figure S2 (Supplementary Materials). Thus, it was possible to calculate the percentage of antiviral released from the nanomicelles for each time interval.

**Figure 9:**
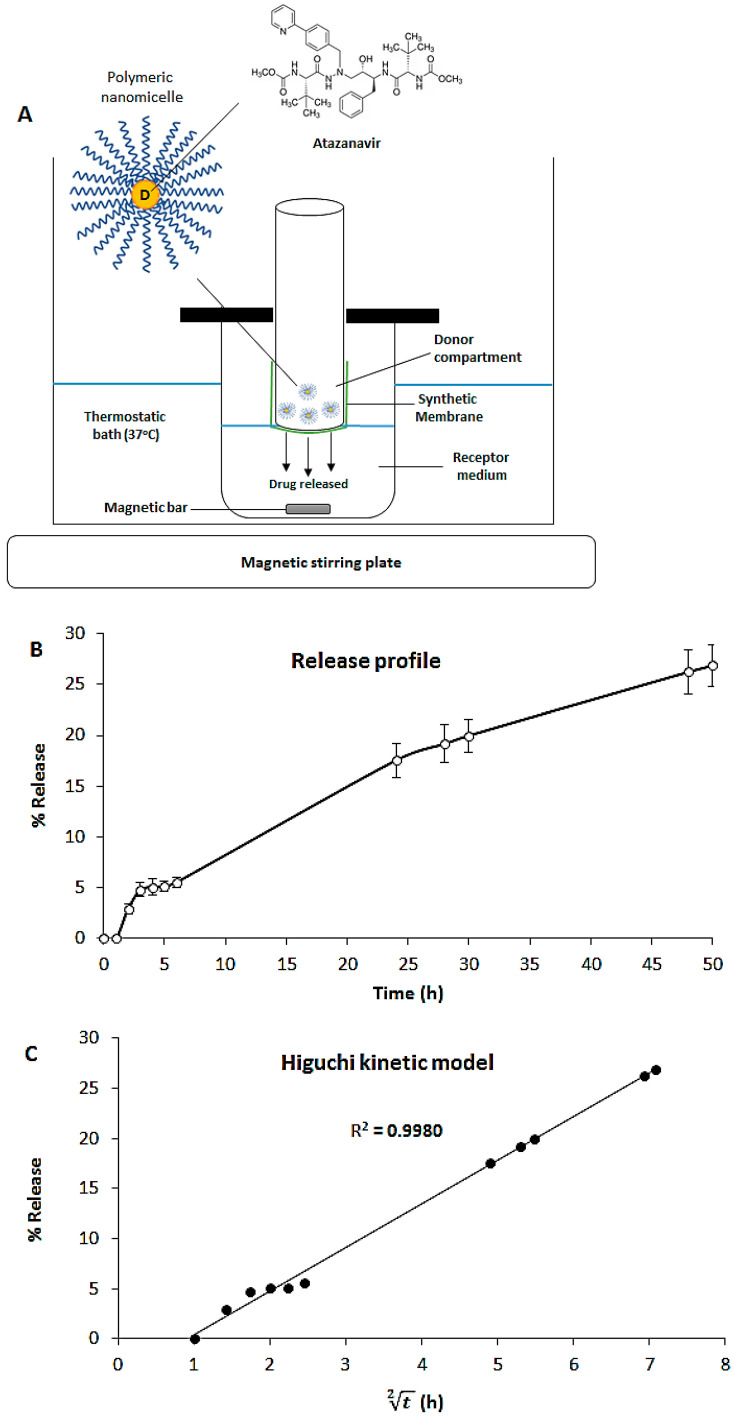
Diffusion cell used in in vitro release studies (**A**); in vitro release profile of atazanavir (ATV) encapsulated in polymeric nanomicelles (**B**); study of ATV kinetics obtained after application of the “Higuchi Model” (**C**).

Figure 9A shows a schematic representation of the equipment used for the in vitro release studies of ATV from nanomicelles. A cellulose acetate membrane (dialysis tube) separates the donor compartment with ATV loaded in nanomicelles from the receptor compartment. Figure 9B shows the in vitro release profile of ATV from polymeric nanomicelles. The ATV release was slow and sustained for 50 h. A total of 26.9 ± 2.2% of the antiviral was released in 50 h without the “burst” effect. The polymeric nanomicelles act as a nanocarrier capable of transporting the antiviral in biological media with slow release.

Figure 9C shows the graph obtained with the Higuchi model. This model is based on the physicochemical principle of diffusion [25,26]; thus, the ATV release is slow, sustained, and controlled by diffusion. Two kinetic models were used to understand the process that controls the release of the antiviral from the nanocarrier. The models applied are the “Zero Order” and “Higuchi Model” [25,26]. In the “Zero Order” model, the release data were directly linearized to obtain the coefficient of determination (R^2^). In the “Higuchi Model”, the square root of time ($\sqrt[{2}]{t}$) was initially calculated with subsequent linearization to obtain the coefficient of determination (R^2^). The coefficients of determination (R^2^) obtained were R^2^ = 0.9657 for the “Zero Order” model and R^2^ = 0.9980 for the “Higuchi Model”. The “Higuchi Model” better fit the release profile of ATV from polymeric nanomicelles with a higher coefficient of determination than the “Zero Order” model (Figure 9C).

## 4. Conclusions

Overall, our results reinforce the potential of Pluronic F127-based micelles as a promising nanotechnology-driven antiviral strategy. Their ability to inhibit SARS-CoV-2 replication, along with their low cytotoxicity and immunomodulatory properties, highlights their applicability in antiviral therapy. Future studies should also explore whether these nanomicelles modulate viral entry by altering host cell membrane properties, including the expression of viral receptors such as ACE2 and TMPRSS2, as well as membrane fluidity. Further in vivo studies and clinical evaluations will be necessary to elucidate the full potential of these nanocarriers in treating COVID-19 and other viral infections.

## Data Availability

The original contributions presented in this study are included in the article/Supplementary Materials. Further inquiries can be directed to the corresponding authors.

## Supplementary Materials

The following supporting information can be downloaded at: https://www.mdpi.com/article/10.3390/v17040518/s1, Figure S1: Zeta Potential determination graph, Figure S2: Analytical curve of atazanavir.
