# Supplementary material for: Nanotechnology-Driven Strategy Against SARS-CoV-2: Pluronic F127-Based Nanomicelles with or Without Atazanavir Reduce Viral Replication in Calu-3 Cells
Source: Viruses. 2025 Apr 1;17(4):518. doi: 10.3390/v17040518 (PMC12031194; doi:10.3390/v17040518)

Supplementary Materials:

Figure S1: Zeta Potential determination graph

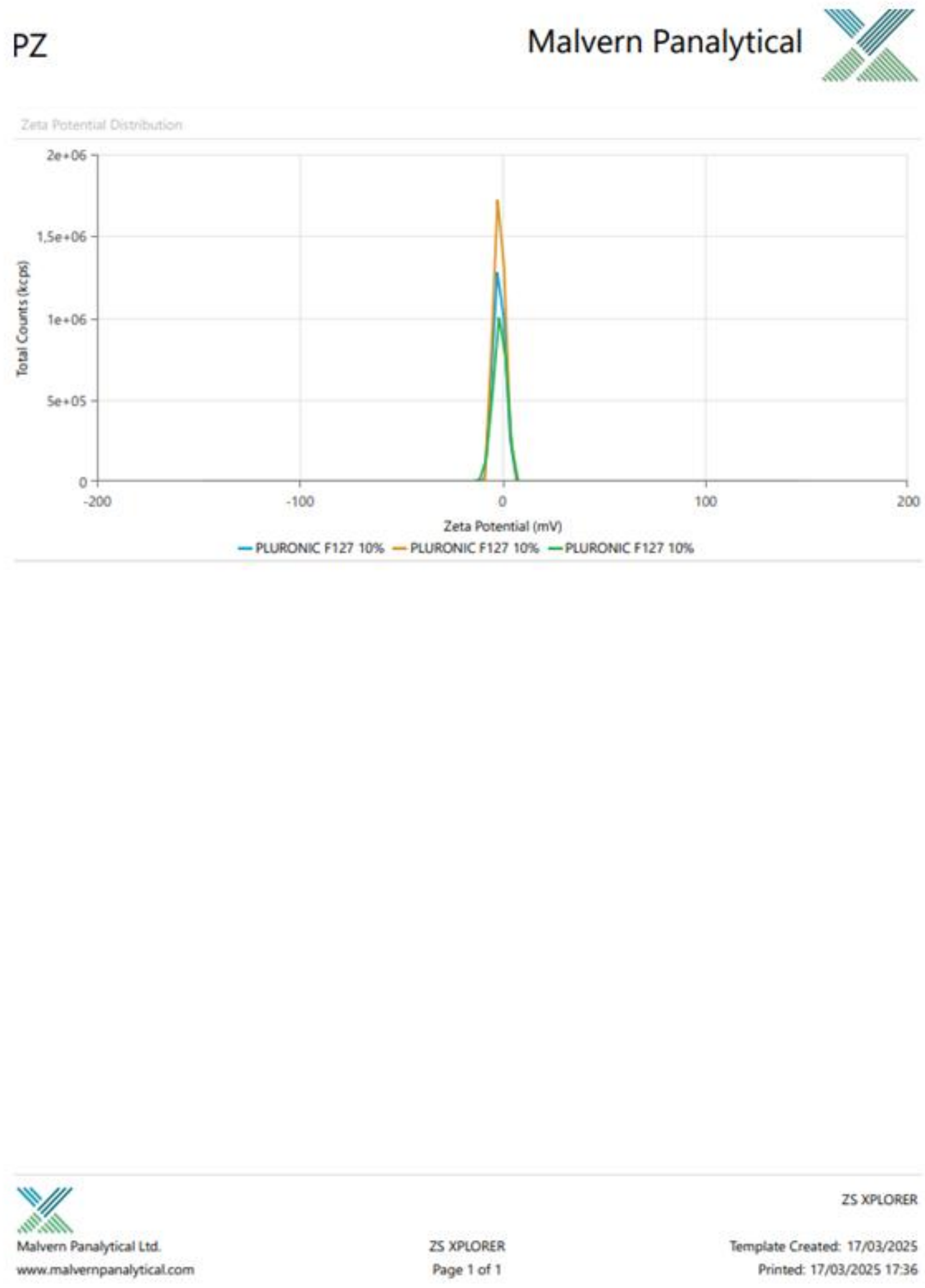

Figure S2: Analytical curve of atazanavir.

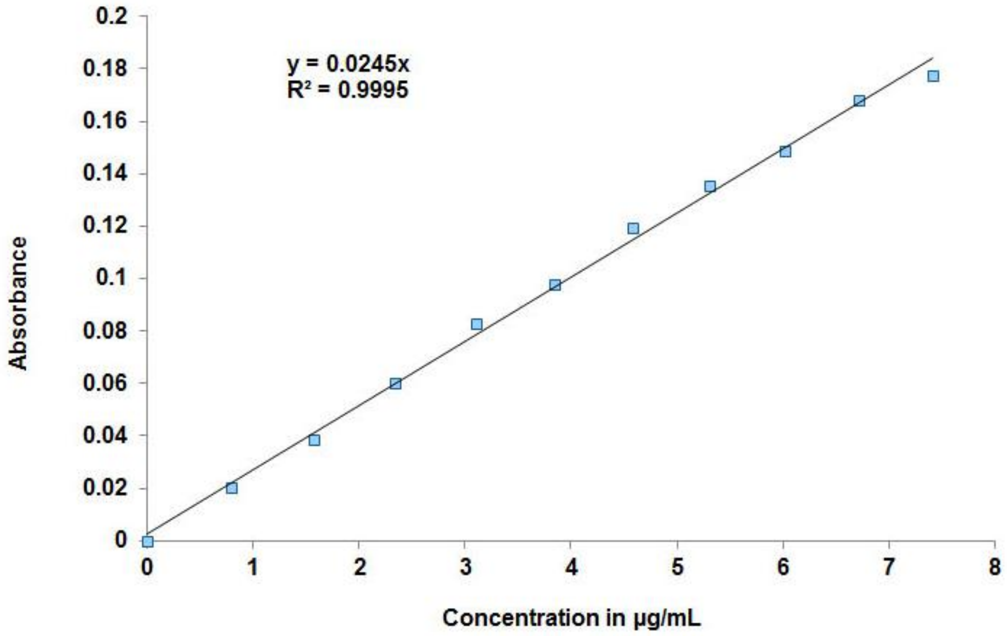

Supplement: Supplementary file 1 [file viruses-17-00518-s001.zip › viruses-3522698-supplementary.pdf]
